# Poor Perinatal Care Practices in Urban Slums: Possible Role of Social Mobilization Networks

**DOI:** 10.4103/0970-0218.51229

**Published:** 2009-04

**Authors:** Zulfia Khan, Saira Mehnaz, Najam Khalique, Mohd Athar Ansari, Abdul Razzaque Siddiqui

**Affiliations:** Department of Community Medicine, J. N. Medical College, A.M.U., Aligarh, India

**Keywords:** Barriers to utilization of services, urban slums, unsafe delivery practices

## Abstract

**Background:**

Making perinatal care accessible to women in marginalized periurban areas poses a public health problem. Many women do not utilize institutional care in spite of physical accessibility. Home-based care by traditional birth attendants (TBA) is hazardous. Inappropriate early neonatal feeding practices are common. Many barriers to perinatal care can be overcome by social mobilization and capacity building at the community level.

**Objectives:**

To determine the existing perinatal practices in an urban slum and to identify barriers to utilization of health services by mothers.

**Study Design:**

This is a cross-sectional descriptive study.

**Setting and Participants:**

The high-risk periurban areas of Nabi Nagar, Aligarh has a population of 40,000 living in 5,480 households. Mothers delivering babies in September 2007 were identified from records of social mobilization workers (Community Mobilization Coordinators or CMCs) already working in an NGO in the area. A total of 92 mothers were interviewed at home. Current perinatal practices and reasons for utilizing or not utilizing health services were the topics of inquiry.

**Statistical Analysis:**

Data was tabulated and analyzed using SPSS 12.

**Results:**

Analyses revealed that 80.4% of mothers had received antenatal care. However, this did not translate into safe delivery practices as more than 60% of the women had home deliveries conducted by traditional untrained or trained birth attendants. Reasons for preferring home deliveries were mostly tradition (41.9%) or related to economics (30.7%). A total of 56% of the deliveries were conducted in the squatting position and in 25% of the cases, the umbilical cord was cut using the edge of a broken cup. Although breast-feeding was universal, inappropriate early neonatal feeding practices were common. Prelacteal feeds were given to nearly 50% of the babies and feeding was delayed beyond 24 hours in 8% of the cases. Several mothers had breastfeeding problems.

**Conclusion:**

Barriers to utilization of available services leads to hazardous perinatal practices in urban slums.

## Introduction

Perinatal care has a tremendous impact on the health of the mother and child.(1–4) However, good quality perinatal care is not uniformly distributed in society.(5) Advancements in technology have made sophisticated tertiary care available to those who can pay. At the same time, the gap between the rich communities and the poor, marginalized, and underserved communities is increasing.(6,7)

Even after reproductive and child health – 2 (RCH-2), it has not been possible to reach a large segment of the marginalized population through the organized health sector.(5) Rapid urban development is outstripping the meager resources at the local municipality level. Even where facilities exist, socio-economic and cultural barriers prevent their optimum utilization by the women who need them most,(8,9) consequently resulting in hazardous health practices.(10,11)

### Objectives

This cross-sectional study was conducted for the following reasons:

- To determine the existing perinatal practices in an urban slum
- To identify barriers to utilization of available services by mothers

## Materials and Methods

*Study area:* This cross-sectional study was conducted in the periurban area of Nabi Nagar, which has a total population of 40,000 living in 5,480 households. This highly congested area is situated on the outskirts of Aligarh, has unplanned houses, few roads, open drains, and no piped water. Nearly one third of the families have moved in from neighboring rural areas within the last 5 years. Most earning members are laborers and small vendors or shopkeepers. A few houses belong to retired government employees.

One Urban Health Post is within 1 km and the Medical College Hospital is within 2 km of the area. There is one Private Maternity Home and 4 Private Clinics in the area.

The Department of Community Medicine at J.N. Medical Collage, AMU runs a health clinic providing pediatric services and routine immunization services in the area. The Social Mobilization Network (SM Net) of UNICEF has a cadre of Community Mobilization Coordinators (CMCs) in the area.

Before starting the study, approval from the institutional ethical committee was obtained.

All live births are routinely recorded by the CMC of the area. A list of 98 families with live births in September 2007 was obtained from the CMC of the area (giving a birth rate of 29.4 per 1000 population). Of these, one mother refused to participate and 5 mothers were not available at home on two visits. In all, 92 mothers were included in the study.

Mothers were visited at home by a female researcher within the first month of delivery and interviewed in a non formal and non judgmental manner, after obtaining informed consent. Information was obtained about antenatal care received, delivery practices, and current newborn feeding practices. Appropriate counseling, treatment, and referral were given wherever needed.

The data was recorded on a preformed interview schedule and coded and analyzed using SPSS 13. The Z test for proportions was used to test the difference between home and institutional deliveries. A value of 0.05 was considered as significant.

## Results

*Socio-cultural profile of the study population:* All mothers had poor socio-economic status and lived in a congested, unsanitary environment in the slum.

As can be seen, from Table 1, a majority of women (67%) preferred to have the delivery at home. A majority were young mothers (84.7% being 30 years or younger), illiterate (56.5%) or just literate (17.4%), and living in unitary families (60.9%) [Table 1].

**Table 1:** Socio-economic variables of the study population

	Home		Institution		Total	
			
	No.	%	No.	%	No.	%
Type of family						
Unitary	39	62.9	17	56.7	56	60.9
Joint	23	37.1	13	43.3	36	39.1
Total	62	67.4	30	32.6	92	100
Age of mother (yrs.)					
20-30	51	82.3	27	90.0	78	84.8
31-40	11	17.7	3	10.0	14	15.2
Total	62	67.4	30	32.6	92	100
Education of mother						
Illiterate and just literate	49	79.0	19	63.3	68	73.9
Literate	13	21.0	11	36.7	24	26.1
Total	62	67.4	30	32.6	92	100
Decision for place of delivery taken by						
Husband	18	29.0	14	46.7	32	34.8
Mother in-law	17	27.4	5	16.7	22	23.9
Self	7	11.3	7	23.3	14	15.2
More than one person	20	32.3	4	13.3	24	26.1
Total	62	67.4	30	32.6	92	100

The decision about the place of delivery was taken by the husband (29%), mother in law (27.4%), or jointly by the family (32.3%).

### Existing Perinatal Practices

*Antenatal care:* A total of 19.6% of the mothers did not receive any antenatal care [Table 2]. Of these, a large majority were those who preferred to deliver at home. Only two women who did not receive any antenatal care delivered in the hospital due to some unforeseen complication of the delivery.

**Table 2:** Antenatal practices in the study population

	Home delivery		Institutional delivery		Total		Z test	
				
	No.	%	No.	%	No.	%	
Any antenatal care received							
No	16	25.8	2	6.7	18	19.6	P<0.5 significant
Yes	46	74.2	28	93.3	74	80.4	significant
Total	62	100	30	100	92	100	-
No. of tet vac. received						
Zero	16	25.8	2	6.7	18	19.6	P<0.5 significant
One	2	3.2	0	0	2	2.2	No comparison
Two	44	71.0	28	93.3	72	78.2	P<0.5 significant
Total	62	100	30	100	92	100	-

There was a significant difference in the rate of antenatal care received (at least one visit and I tetanus toxoid) among women who delivered at home and those who delivered in an institution. While overall a majority of women (80.4%) did have at least one antenatal check-up either in government hospital or a private clinic/nursing home, the number was significantly higher in those women who had institutional deliveries (z=2.145.Sig.)

A total of 9.6% of the mothers did not receive a dose of Inj. Tetvac. These were the same mothers who did not receive an antenatal check-up. The majority of women received two doses of Inj. Tetvac (78.2%). However, the rate was significantly higher in women who chose to have an institutional delivery (z=2.477.Sig).

*Hazardous practices in home delivery:* Natal care was found to be poor in the case of deliveries at home [Table 3].

**Table 3:** Hazardous practices in home delivery

	No.	%
Delivery conducted by		
Trained dai	31	50.0
Untrained dai	25	40.3
Nurse	6	9.7
Total	62	100
Position of conducting delivery		
Squatting	35	56.5
Lying down	27	43.5
Total	62	100
Umbilical cord cut by		
New razor blade	25	40.3
Edge of broken cup	37	59.7
Total	62	100
Complications from delivery		
No	59	95.2
Yes	3	4.8
Total	62	100

*Delivery attended by:* Fifty percent of the home deliveries were attended by Trained Birth Attendants and 40% were attended by Untrained Birth Attendants. A private nurse attended 10% of the home deliveries.

*Position:* A majority of women delivered their babies in the squatting position (56.5%).

*Cord cut by:* The umbilical cord was cut by a new blade in 59.9% of the cases but by traditional objects such as the edge of a broken cup in 40.3% of the cases.

*Complications of delivery:* While a majority of the women had normal deliveries, 4.8% said they had some complication during delivery such as prolonged labor.

*Feeding practices:* All newborns were breast-fed [Table 4].

**Table 4:** Newborn feeding practices

	Home delivery		Institution delivery		Total		Z test
				
	No.	%	No.	%	No.	%	
Infant feeding							
Breast milk	62	100	30	100	92	100	No comparison
Top feeds	0	0	0	0	0	0	
Total	62	100	30	100	92	100	
Colostrum given to baby						
No	21	33.9	3	10	24	26.1	P<0.5 significant
Yes	41	66.1	27	90	68	73.9	P<0.5 significant
Total	62	100	30	100	92	100	
Time of giving first feed						
Within 6 hrs. of birth	29	46.8	20	66.7	49	53.2	Not significant
Within 6-12 hrs. of birth	14	22.6	6	20.0	20	21.7	Not significant
Within 12-24 hrs. of birth	5	8.1	1	3.3	6	6.5	Not significant
More than 24 hrs.	14	22.6	3	10.0	17	18.4	Not significant
Total	62	100	30	100	92	100
Prelacteal feeds						
Given	31	50	11	36.7	42	45.7	Not significant
Not given	31	50	19	63.3	50	54.3	Not significant
Total	62	100	30	100	92	100
Feeding problems						
No	57	91.9	24	80.0	81	88.1	Not significant
Yes	5	8.1	6	20.0	11	11.9	Not significant
Total	62	100	30	100	92	100	

*Colostrum:* Colostrum was given to 73.9% of the babies and discarded in the rest.

*First feed:* The first feed was given within 6 hours of birth in 66.7% of the newborns in institutional delivery compared with 46.8% in home delivery. Overall, 53.2% of the newborns were breast-fed within 6 hours of birth. However, the time of giving first feed was delayed beyond 24 hours of birth in 18.4% of the cases, mostly in home delivery (22.6%) compared with institutional delivery (10.0%).

*Prelacteal feeds:* Prelacteal feed such as ‘gutti’ or ‘pehua’ was given to 45.7% of the babies.

*Breastfeeding problems:* A total of 11.9% of the mothers were having some breast-feeding problems at the time of the survey.

### Barriers to Utilization of Health Services

*Reasons for choosing home or institution for delivery:* Table 5 shows the main reasons stated by the mothers for choosing home delivery (n=62) or institutional delivery (n=30).

**Table 5:** Reasons for choosing place of delivery

	No.	%
Home delivery (n = 62)		
Tradition	26	41.9
Economic reason	19	30.7
Rude behavior of personnel in hospital	4	6.5
Nobody to take care of home	8	12.9
Fear of hospital	3	4.8
Other	2	3.2
Govt. hospital (*n* = 22)		
Close to home	8	36.4
Complication in earlier pregnancy	6	27.3
Proper attention given	5	22.7
Sympathetic attitude of health personnel	0	0
Other	3	13.6
Private nursing home (n = 8)		
Close to home	2	25.0
Complication in earlier pregnancy	1	12.5
Proper attention given	3	37.5
Sympathetic attitude of health personnel	2	25.0
Other	0	0

*Home delivery:* Most mothers said that giving birth at home with assistance from a traditional attendant was a norm followed in their family and society. Childbirth being a natural process, there was no need to change the norm. Thus, the most common reason for home delivery was stated as being family tradition (42%). Economic constraint was also a common reason for avoiding institutional delivery, as even in the government hospitals medicines and investigations were not free (30%). Other reasons for preferring home delivery included rude behavior of hospital personnel (7%), nobody to take care of the home during their absence (13%), fear of hospitals (4.8%), and other reasons (3.2%).

*Institutional delivery:* A total of 33% of the women delivered in an institution, either a government hospital (n=22) or a private nursing home (n=8). The main reasons stated for choosing a government hospital were its proximity to home, and a history of complications in earlier pregnancies. The main reasons for choosing a private nursing home were proximity to home and the perception that proper attention will be given to the patient.

Thus, the important barriers to utilization that have been identified include a strong tradition of home delivery and economic constraints of the family.

## Discussion

This study highlights that in spite of health services being within reach, a majority of women choose to deliver at home, often by untrained birth attendants. Hazardous delivery practices and undesirable feeding practices were common. The major limiting factors for institutional delivery were family tradition and economic constraints.

There is evidence to show that the demand side barriers to access of services, such as tradition, lack of knowledge, and financial constraints may be as important as supply factors in deterring patients form utilizing services.(5,8,9)

This study shows that good quality Maternal and Child Health (MCH) services are not reaching those who need them most.

Thus, although at least one antenatal visit was availed by 80% of the mothers, this did not translate into good delivery practices for most, as 67% mothers preferred to deliver at home. An earlier study in a periurban area of east Delhi(11) has also reported an ANC utilization rate of 74.3%. However, this is more than the overall ANC coverage rate in Uttar Pradesh, which is 25%.(12)

The rate of delivery at home is 67% in the present study, which is less than 73% reported for all of Uttar Pradesh.(12) A study from urban slums and periurban areas in Delhi has reported 70% home deliveries of which 81.9% were attended by untrained dais.(11) A community-based case control study in Delhi slums has also reported that most deliveries were conducted at home by untrained dais.(13) The rate of births attended by untrained birth attendants is comparatively lower in the present study (40.3%).

Delivering a baby in the squatting position is beneficial in that it shortens the duration of the 1^st^ and 2^nd^ stages of labor. However, without proper perineal support provided by birthing chairs or cushions, the incidence of maternal injury such as perineal tear is very high. Given the current delivery practices at home, it is thus considered to be hazardous for the mother.(14)

Although breast feeding is the norm, giving prelacteal feeds is a deep-rooted custom in India and many studies have reported up to 100% of mothers giving prelacteal feeds.(14,15) However, in this study, 45.7% of the mothers gave prelacteal feeds and the rate was not significantly different in home deliveries compared with institutional deliveries. Delaying of the first feed (22.6%) and discarding of colostrums (26.1%) were other customs that have been reported in similar earlier studies.(13–17)

Socioeconomic barriers to utilization of services are important.

For many of these urban families, pregnancy and childbirth is not a priority. The tradition of home deliver with help from untrained birth attendants is the societal norm. However, antenatal care and Inj. Tetvac is well accepted by the majority.

An important barrier to acceptance of services is economic constrain. A total of 30% of the families from the urban slum in this study said they were unable to afford services even in government hospitals because of the cost of medicines and investigations, even though consultation was free. They visited hospitals because of complications during delivery in the current or an earlier pregnancy. Evidence suggests that demand side barriers such as cost of services are important barriers to obtaining services, especially in poor and vulnerable groups.(8) In a study on barriers to access of health services in Bangladesh, 45% of the women stated financial reasons for not accessing health services.(18) However, a study in Maharashtra, India points out that women were able to overcome the economic constrains if they felt that services outweighed the cost.(9)

It may be possible to overcome the economic constraints by ensuring availability of trained birth attendants for home delivery in urban settings as in rural setting. There is an ongoing debate about reinforcing home-based birthing strategies with skilled birth attendants in developing countries.(18) Trained birth attendants are more likely to use clean delivery practices compared with untrained birth attendants. However, a study from rural Bangladesh has shown no significant difference in the level of post-partum infection rate in deliveries conducted by trained birth attendants compared with untrained birth attendants.(19) Thus, it is logical that because institutional facilities are available in urban areas, under the RCH 2 program, women, especially in urban areas, are encouraged to have their deliveries in an institution.

In this study, 33% of the women who delivered in institutions, either government or private, had significantly better indicators of maternal health care, such as utilization of antenatal care and appropriate infant feeding practices, compared with those mothers who delivered at home. Thus, it is justifiable to prioritize the promotion of institutional deliveries for the mothers in urban slum dwellings.

Social mobilization, using a participatory approach is effective in improving perinatal health indicators.(20) In the study area for the present survey, the community-based social mobilization program has effectively improved participation in the National Polio Eradication program to almost 100%. Proper monitoring and ongoing evaluation and using flexible guidelines has made this program exceptionally effective.(21) The NGO already working in the area of child health can easily expand its message to include perinatal care, perhaps without incurring any additional costs.

**Limitations of the study:** Being a pilot study, the area covered is relatively small but typical of most periurban slum areas. Utilization of government services (at the Medical College Hospital) may be more than usual for such underserved areas because of its proximity and because of the referrals from the Health Clinic.

## Conclusion

Physical accessibly to services does not necessarily lead to service utilization. Social and cultural accessibility is as important as physical accessibility. Important barriers to service utilization in this study include the tradition of home delivery and economic constraints.

Counseling and social mobilization and can remove the traditional barriers to a large extent in health seeking behavior.(10,21,22)

## Recommendations

Perhaps the NGOs already working in urban areas for other projects can be utilized to provide information education and communication support for maternal and newborn care. At the same time, care must be taken to meet the demand generated by social mobilization.

Any upgrading of services as planned under RCH 2 must go hand in hand with community-based research at the local level and address the barriers to community acceptability.

To overcome the economic barriers, further studies are recommended to study the role of health insurance and micro credit schemes on service utilization.
